# A Two-Stage Time-Domain Equalization Method for Mitigating Nonlinear Distortion in Single-Carrier THz Communication Systems

**DOI:** 10.3390/s25154825

**Published:** 2025-08-06

**Authors:** Yunchuan Liu, Hongcheng Yang, Ziqi Liu, Minghan Jia, Shang Li, Jiajie Li, Jingsuo He, Zhe Yang, Cunlin Zhang

**Affiliations:** 1 Key Laboratory of Terahertz Optoelectronics, Ministry of Education, Beijing 100048, China; 2230602063@cnu.edu.cn (Y.L.); 2240601005@cnu.edu.cn (H.Y.); 2230602033@cnu.edu.cn (Z.L.); 2240602065@cnu.edu.cn (M.J.); 2240602066@cnu.edu.cn (S.L.); 2240602041@cnu.edu.cn (J.L.); yangzhe7010@cnu.edu.cn (Z.Y.); 3827@cnu.edu.cn (C.Z.); 2 Beijing Key Laboratory for Terahertz Spectroscopy and Imaging, Beijing 100048, China; 3 Beijing Advanced Innovation Center for Imaging Theory and Technology, Beijing 100048, China; 4 Department of Physics, Capital Normal University, Beijing 100048, China

**Keywords:** deep learning, terahertz, digital communication, LSTM

## Abstract

Terahertz (THz) communication is regarded as a key technology for achieving high-speed data transmission and wireless communication due to its ultra-high frequency and large bandwidth characteristics. In this study, we focus on a single-carrier THz communication system and propose a two-stage deep learning-based time-domain equalization method, specifically designed to mitigate the nonlinear distortions in such systems, thereby enhancing communication reliability and performance. The method adopts a progressive learning strategy, whereby global characteristics are initially captured before progressing to local levels. This enables the effective identification and equalization of channel characteristics, particularly in the mitigation of nonlinear distortion and random interference, which can otherwise negatively impact communication quality. In an experimental setting at a frequency of 230 GHz and a channel distance of 2.1 m, this method demonstrated a substantial reduction in the system’s bit error rate (BER), exhibiting particularly noteworthy performance enhancements in comparison to before equalization. To validate the model’s generalization capability, data collection and testing were also conducted at a frequency of 310 GHz and a channel distance of 1.5 m. Experimental results show that the proposed time-domain equalizer, trained using the two-stage DL framework, achieved significant BER reductions of approximately 92.15% at 230 GHz (2.1 m) and 83.33% at 310 GHz (1.5 m), compared to the system’s performance prior to equalization. The method exhibits stable performance under varying conditions, supporting its use in future THz communication studies.

## 1. Introduction

Terahertz communications have attracted widespread attention in recent years due to their ultra-high frequency and extremely wide bandwidth [1]. Typically operating in the frequency range of 0.1 to 10 THz, THz systems offer abundant spectrum resources, theoretically enabling transmission rates of hundreds of Gbps to Tbps. Thus, THz communication is considered a key enabler for next-generation wireless systems [2]. However, severe free-space path loss and strong molecular absorption—particularly by water vapor and oxygen—lead to rapid signal attenuation [3,4,5,6,7,8]. Additionally, multipath fading and the non-ideal behaviors of high-speed front-end components such as amplifiers, mixers, and antennas introduce significant nonlinear distortion and phase noise [9,10,11,12]. These impairments result in highly distorted and complex channel responses, drastically degrading system performance [13,14,15]. Therefore, a powerful equalization technique is critical for reliable THz communications [16].

Conventional channel equalization methods struggle to cope with these challenges. Linear equalizers such as minimum mean square error (MMSE) offer low complexity but fall short in addressing nonlinear impairments such as amplifier saturation and frequency-dependent phase distortion [17]. Nonlinear compensation techniques, such as digital predistortion (DPD), often rely on forward modeling and adaptive feedback, but are difficult to implement efficiently in THz systems due to hardware constraints and rapidly varying channel dynamics [18]. In recent years, with the increasing application of DL in physical-layer communications, many researchers have proposed deep neural network (DNN)-based equalization methods to overcome the limitations of traditional algorithms in modeling accuracy and nonlinear processing [19,20,21,22,23]. Leveraging the powerful fitting capability of DL, DNNs have become a promising solution for THz systems [24,25,26]. Some recent studies have applied DNN-based equalizers to address nonlinear issues in THz links. For instance, Wang et al. proposed a time–frequency domain equalizer (TFDE) based on a 2D convolutional neural network (Conv2D) to mitigate linear and nonlinear impairments in RoF-OFDM systems [27]. Shi et al. introduced an end-to-end waveform-to-waveform auto equalization framework (W2WAEF) to address similar challenges in seamlessly integrated fiber–THz communication systems [28].

In this work, a two-stage DL equalization method for single-carrier signals in the time-domain is proposed to effectively solve the serious nonlinear distortion problem in terahertz systems. The method hierarchically models the time-domain features of the received signal and realizes the joint compensation of multi-scale nonlinear effects. Specifically, in the first stage, the transmitted and received signals are upsampled to enable high temporal resolution, supporting global feature modeling in the first stage; then, they are downsampled in the second stage to focus on the compact refinement of residual distortion. To the best of our knowledge, this is the first time that this “coarse-to-fine” advanced learning strategy is systematically introduced into the equalization framework in a single-carrier terahertz communication system through explicit resolution control (up/downsampling) and stage division. Two DNN models are cascaded to form a complete equalizer to gradually suppress the nonlinearity in the received signal. Compared with the single-stage time-domain equalizer, the proposed two-stage scheme has an increased computational complexity, but significantly improves the processing capability, especially for effectively compensating for the severe nonlinear distortion that is difficult to handle with the single-stage model. Both simulation and measured results show that this scheme significantly reduces the system BER, demonstrates excellent nonlinear distortion suppression capability and enhanced system robustness, and provides an effective new approach to solving the equalization problem under complex channel conditions in terahertz communications.

## 2. Two-Stage Time-Domain Equalization Method

**Figure 1:**
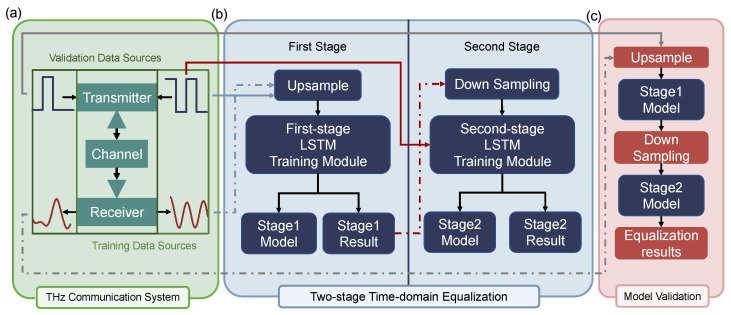
Two-stage time-domain equalization structure. Architecture of the proposed two-stage time-domain equalization method. (**a**) THz communication system model and dataset construction. Two datasets under different frequencies and transmission distances are collected as training and validation data sources. (**b**) Training process of two-stage equalizer. Both the transmitted and received signals are upsampled before being processed by the first-stage long short-term memory (LSTM)-based equalizer, making it more effective in capturing nonlinear distortions. The output is then downsampled and refined by the second-stage LSTM equalizer. (**c**) In the inference stage, the trained Stage-1 and Stage-2 models are cascaded to process validation data and generate equalization results.

As shown in Figure 1, the DL-based two-stage time-domain equalization structure proposed in this study consists of three main components: the THz wireless communication system, the DL-based two-stage time-domain equalization module, and the equalization model validation module.

### 2.1. THz Wireless Communication System

In the THz wireless communication system, a pseudo-random bit sequence is first encoded using low-density parity-check (LDPC) coding and modulated using quadrature phase-shift keying (QPSK). The resulting symbols are then mapped to baseband In-phase and Quadrature (I/Q) signals via a field-programmable gate array (FPGA) and a digital-to-analog converter (DAC). Subsequently, the baseband signal is upconverted to the target carrier frequency through a radio frequency (RF) chain for transmission. At the receiver side, the signal is first downconverted to an intermediate frequency (IF) using a downconversion module, amplified by an intermediate-frequency low-noise amplifier (LNA), and further downconverted to baseband. The baseband signal is then digitized using an analog-to-digital converter (ADC) and processed by an FPGA. Frame synchronization is performed to extract valid segments for DL. After synchronization, the signal is fed into the proposed two-stage DL-based equalization module. Due to the highly directional nature and narrow beamwidth of THz signals, and given that the experiments are conducted under short-range line-of-sight (LOS) conditions, the effect of multipath interference is considered negligible [29,30,31,32]. It is also important to note that the selected channel is far from molecular resonance absorption frequencies, and the communication distance is relatively short [33,34,35]; thus, in this study, the dominant source of nonlinearity is attributed to the THz hardware itself.

### 2.2. Long Short-Term Memory

In our proposed two-stage time-domain equalization module, we use an LSTM network to deal with nonlinear noise and other distortions in terahertz communication systems. LSTM networks have been widely used in time series processing research, and their excellent performance has been fully verified and recognized.

**Figure 2:**
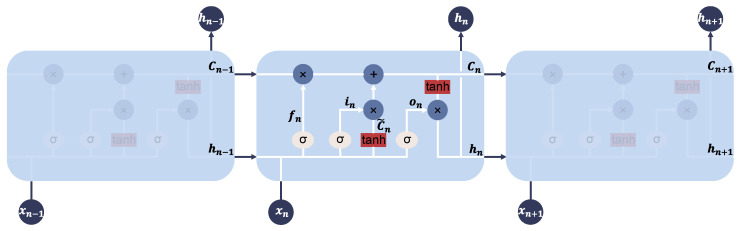
LSTM block diagram at time step *n*: input vector $x_{n}$, current cell state $C_{n}$, candidate cell state ${\tilde{C}}_{n}$, hidden state $h_{n}$, and forget gate $f_{n}$, input gate $i_{n}$, and output gate $o_{n}$.

Figure 2 illustrates the structure of the LSTM. As shown in the figure, at time step *n*, the LSTM module consists of an input vector $x_{n}$, current cell state $C_{n}$, candidate cell state ${\tilde{C}}_{n}$, hidden state $h_{n}$, and the forget gate $f_{n}$, input gate $i_{n}$, and output gate $o_{n}$. The core working mechanism of the LSTM is to forget irrelevant information while retaining useful information. At each time step, the hidden state $h_{n}$ is output. During the establishment of the two-stage time-domain equalization model, the input sequence $X=\{x_{1},\ldots ,x_{N}\}$ produces the output sequence $H=\{h_{1},\ldots ,h_{N}\}$ through the following equations and is processed iteratively.

Forget gate:

(1) $$f_{n}=\sigma \left(W_{f}\cdot \left[h_{n-1},x_{n}\right]+b_{f}\right).$$

Memory gate:

(2) $$i_{n}=\sigma \left(W_{i}\cdot \left[h_{n-1},x_{n}\right]+b_{i}\right).$$

Temporary state of the cell:

(3) $${\tilde{C}}_{n}=\tanh\left(W_{c}\cdot \left[h_{n-1},x_{n}\right]+b_{c}\right).$$

Current state of the cell:

(4) $$C_{n}=f_{n}*C_{n-1}+i_{n}*{\tilde{C}}_{n}.$$

Output gate:

(5) $$o_{n}=\sigma \left(W_{o}\cdot \left[h_{n-1},x_{n}\right]+b_{o}\right).$$

State of hidden layer:

(6) $$h_{n}=o_{n}*\tanh\left(C_{n}\right).$$

Here, *W* represents the weight matrix, *b* denotes the bias vector, and σ is the sigmoid activation function. $h_{n-1}$ and $C_{n-1}$ are the hidden state and cell state from the previous time step n−1, while $h_{n}$ and $C_{n}$ are the newly generated states at the current time step, which will be passed on to the next time step n+1. Therefore, LSTM not only accumulates information over a long period but also has the ability to reset old states through the forget gate, allowing it to restart the accumulation of information when needed.

It should be noted that although we used an LSTM network to solve the nonlinear noise problem in terahertz communication systems in this study, the choice of network architecture is not the focus of this paper. The reason for choosing LSTM is that it performs well in most time series tasks [36,37,38,39,40,41,42]. We use the network model as a tool to address the distortion problem in terahertz communication systems. The innovation of this paper lies in the two-stage time-domain equalization training strategy, so this paper will not discuss the choice of network architecture in detail.

### 2.3. Two-Stage Time-Domain Equalization Method

As shown in Figure 1, the proposed two-stage DL equalizer employs a global–local cooperative strategy to model and compensate for nonlinear distortion in THz communication systems. Figure 1a illustrates the process of data acquisition and construction. The system collects two datasets: training data and validation data. These two sets differ in frequency and transmission distance, and are used to evaluate the robustness and generalization ability of the model.

As depicted in Figure 1b, during the training phase, the collected transmit and receive signals are first upsampled and then sequentially processed by two DL modules. The first stage (global equalization module) performs global feature learning enabled by high temporal resolution through upsampling to capture broad nonlinear distortions. Its output is then downsampled and fed into the second stage (local equalization module) for localized residual compensation after downsampling. After training, two independent equalizer models are obtained: the global and the local equalizers.

As shown in Figure 1c, during the testing phase, the trained models are cascaded to form a complete equalization structure. The validation data are first upsampled and then passed through the global and local equalization modules sequentially to produce the final equalized signal. The BER is measured to evaluate model robustness under varying communication conditions.

In the global equalization module, signal upsampling enhances temporal resolution, allowing the DL model to capture nonlinear features caused by hardware impairments and complex channel effects. Then, global channel feature learning is performed at this scale.

The output of the global module is then downsampled and processed by a local equalization module to further compensate the signal locally. To maintain low implementation complexity while ensuring temporal awareness, the second stage also adopts an LSTM-based architecture. It is worth emphasizing that the two-stage framework itself is highly flexible, allowing different network structures to be selected for each stage as long as the desired balance effect can be achieved. Although heterogeneous combinations such as “LSTM in the first stage and CNNs in the second stage” can be adopted, we still choose to use LSTM in both stages, mainly for the consideration of unified structural design to avoid the additional design and parameter tuning complexity brought about by the hybrid architecture. By applying targeted residual corrections before decision-making, the local equalizer further improves overall equalization accuracy.

To assess the generalization capability of the proposed equalization architecture, testing data are collected under varying carrier frequencies and LOS distances by adjusting the operating conditions of the THz system. BER comparisons before and after equalization confirm that the two-stage equalizer achieves strong adaptability and robustness across different channel conditions.

### 2.4. Up/Downsampling Method

In this study, we adopted linear interpolation-based upsampling and downsampling methods to control the time resolution of the signal.

In the first-stage equalization, the acquired transmitted and received signals are upsampled before being input into the network for training. The linear interpolation method is employed in the upsampling process. For an original signal x[n], upsampling by a factor of *L* involves two steps: (1) zero insertion, where L−1 zeros are inserted between each pair of adjacent samples; and (2) linear estimation of the intermediate values. The interpolated signal is given by the following formula:

(7) $$x_{\mathrm{up}}\left[k\right]=x\left[n\right]+\frac{k\;\mathrm{mod}\;L}{L}\cdot \left(x\left[n+1\right]-x\left[n\right]\right),\;\mathrm{where}\;k=nL+i,\;i=0,1,\ldots ,L-1.$$

Here, x[n] and x[n+1] denote two adjacent original signal samples. The index *k* refers to the position in the upsampled sequence, while *n* is the corresponding original index such that k=nL+i. The operator mod returns the remainder when *k* is divided by the upsampling factor *L*, and thus, $\frac{k\;\mathrm{mod}\;L}{L}$ represents the relative position between x[n] and x[n+1]. This term acts as a linear interpolation weight. The resulting $x_{\mathrm{up}}\left[k\right]$ is the interpolated value at the *k*-th position of the upsampled signal.

In the second-stage equalization, the coarse equalized signal output by the first-stage equalization model and the transmission signal are downsampled and then the second-stage equalization training is performed. In order to avoid aliasing and retain the waveform trend, we use linear interpolation to reconstruct the continuous approximation of the upsampled output. The signal is then evaluated at a lower rate position to generate a downsampled sequence.

(8) $$x_{\mathrm{down}}\left[m\right]=x\left(mD\right)$$

In this equation, x(t) denotes the continuous signal reconstructed from the original discrete sequence via linear interpolation. The index *m* indicates the position in the downsampled sequence, and *D* is the downsampling factor. The value $x_{\mathrm{down}}\left[m\right]=x\left(mD\right)$ is obtained by evaluating the reconstructed signal at intervals of *D*, thereby producing a lower-resolution version of the original signal while retaining its essential shape.

### 2.5. Frame Synchronization Using Zadoff–Chu Sequences

In order to achieve accurate frame synchronization, a Zadoff–Chu (ZC) sequence is embedded at the beginning of each transmission frame when the required signal is acquired for the experiment. The ZC sequence has constant amplitude and zero cycle autocorrelation, making it very effective in synchronization tasks in wireless communications.

(9) $$z\left[n\right]=\left\{\begin{array}{cc} \exp\left(-j\frac{\pi rn(n+1)}{N}\right), & \mathrm{for}\;N\;\mathrm{odd}\\ \exp\left(-j\frac{\pi rn^{2}}{N}\right), & \mathrm{for}\;N\;\mathrm{even} \end{array}\right.,\;0\leq n<N$$

Here, z[n] represents the *n*-th sample of the ZC sequence, *N* is the sequence length, and *r* is the root index of the sequence, which must be coprime with *N* to ensure ideal correlation properties. The exponential term creates a phase progression that results in a constant-modulus complex-valued sequence. The choice between n(n+1) and $n^{2}$ depends on whether *N* is odd or even.

At the receiver side, we apply a sliding cross-correlation between the incoming signal and the known reference ZC sequence. The frame boundary is identified by the location of the correlation peak, providing precise alignment for subsequent equalization. This synchronization step is implemented in LabVIEW and forms the basis for all downstream processing stages.

### 2.6. Data Preprocessing

In this study, in order to take advantage of LSTM in time series processing, we combined the concept of shift register in a traditional equalizer and used sliding window to preprocess the collected signal. Similar to the shift register, our sliding step is 1 and the time window length is *L*. At the same time, in order to ensure that the amount of data after the sliding window remains unchanged and that the subsequences at the boundary can be fully extracted, we used zero padding to pre-fill L−1 zero-value samples at the front-end of the original transmission signal.

This processing method is structurally consistent with the shift register: the generation process of each new sample is equivalent to moving a fixed-length region backward as a whole by one time unit and inserting the new value at the current moment. After this step, the transmission signal is organized into multiple overlapping subsequences, which can be used for subsequent training of the equalization model.

The processed signal is represented as

(10) $$y=\left[\begin{array}{ccccc} 0 & 0 & \ldots & 0 & y_{1}\\ 0 & 0 & \ldots & y_{1} & y_{2}\\ \vdots & \vdots & \ddots & \vdots & \vdots \\ y_{1} & y_{2} & \ldots & y_{L-1} & y_{L}\\ \vdots & \vdots & \; & \vdots & \vdots \\ y_{N-L+1} & y_{N-L+2} & \ldots & y_{N-1} & y_{N} \end{array}\right]$$

The received signal after data preprocessing corresponds one-to-one with the transmitted signal to form the original dataset. For the two-stage time-domain equalization module, both trainings will go through the data preprocessing part.

### 2.7. Data Augmentation

To enhance the generalization and noise robustness of the model, we perform data augmentation by introducing low signal-to-noise ratio (SNR) training samples. Specifically, the received signal is passed through a simulated additive white Gaussian noise (AWGN) channel to generate a noisy version with an SNR between −5dB and 0dB. These data are then passed through a tanh function to further impose nonlinear effects. The augmented samples are concatenated with the original (high SNR) training data and randomly shuffled to ensure diversity during mini-batch construction. This strategy enables the trained model to better generalize to unknown channel conditions and remain robust under varying degrees of interference.

## 3. Experiment Setup

### 3.1. Experimental System Setup

**Figure 3:**
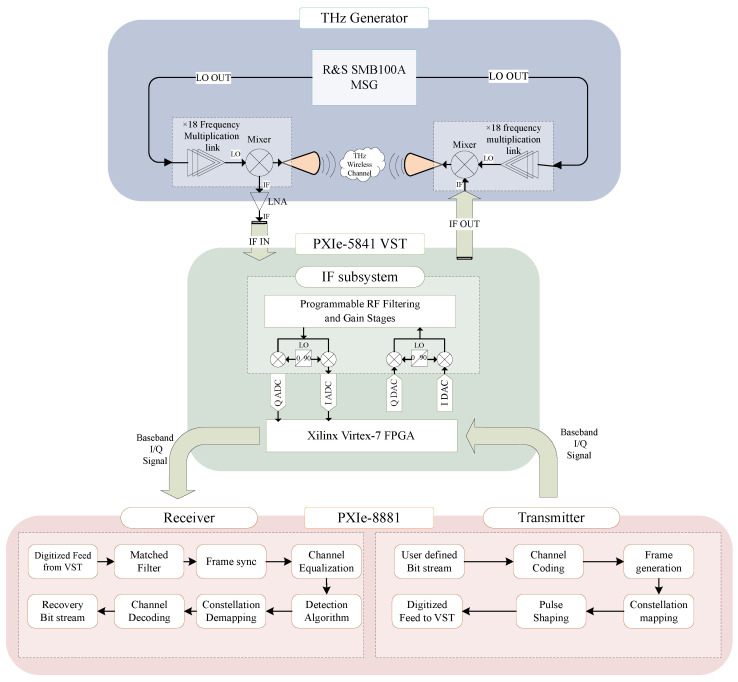
Signal flow chart of the THz wireless communication system performance test platform. The system consists of baseband signal generation and receive signal processing modules (LabVIEW), PXIe-based transceiver (VST), upconversion and downconversion chains based on Schottky diode mixers and multipliers, and a single carrier antenna. The communication link operates under indoor line-of-sight (LoS) conditions in the frequency range of 220 to 330 GHz.

**Figure 4:**
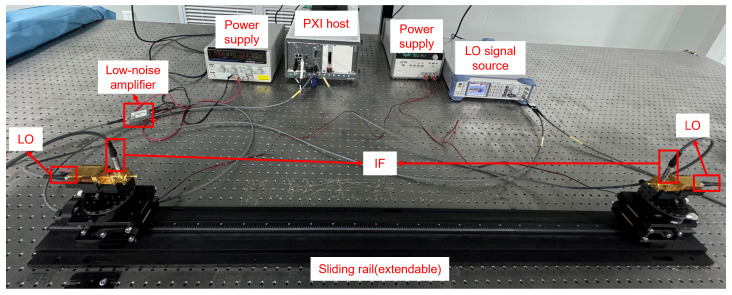
Photograph of the real-time baseband digital signal processing testbed for THz communication. The platform integrates a PXIe-based system for baseband I/Q signal generation and acquisition, a Schottky diode-based RF front-end for up/downconversion, and IF-band low-noise amplification. Key components include a PXIe-8881 embedded controller, PXIe-5841 VST, and external LO and IF circuits. Baseband processing is implemented in LabVIEW 21.0, and equalization is performed offline using Python 3.9.18.

Figure 3 and Figure 4 illustrate the signal flow and the physical layout of the THz wireless communication test platform, respectively. At the transmitter, the upconversion module consists of a frequency multiplier chain (including one frequency doubler and two frequency triplers) and a mixer, both based on Schottky diode technology. The frequency multiplier chain upconverts the local oscillator (LO) signal, with an input power range of 3–6 dBm, to any carrier signal between 220 GHz and 330 GHz. The mixer modulates the upconverted carrier signal with the IF signal carrying the information, which has a maximum input power of −10 dBm, and the double-sideband conversion loss is 10 dB. The maximum output power of the upconversion module is −15 dBm. After generating the terahertz signal, the upconversion module transmits it through a horn antenna with a gain of 20 dBi into the indoor channel.

To generate the local oscillator (LO) signal, the R&S (Rohde & Schwarz, Munich, Germany) SMB100A RF and microwave signal generator (MSG) is used, which can produce highly stable sinusoidal signals in the range of 100 kHz to 40 GHz. The LO signal is then output through a power splitter to the transceiver upconversion module. For generating the intermediate frequency (IF) signal, the PXIe-5841 vector signal transceiver (VST) is employed. This instrument integrates a vector signal generator (RF output path), a vector signal analyzer (RF input path), high-speed serial interfaces, and an FPGA for real-time signal control. The PXIe-5841 combines the flexibility of a software-defined radio (SDR) architecture with the high performance of RF instruments. In this platform, the PXIe-5841 receives baseband I/Q signals from the embedded controller PXIe-8881 through the PXIe bus. These signals are captured by the FPGA and converted via two DAC channels with a maximum sampling rate of 1 GS/s. The signals are then upconverted by the RF output submodule into IF signals with an instantaneous RF bandwidth of up to 1 GHz, covering a range from 125 MHz to 6 GHz.

On the receiving end, the THz signal is downconverted by a downconversion module matching the transmitter’s setup, and the downconverted IF signal is received by the PXIe-5841. The signal is further downconverted to baseband through the corresponding RF input submodule, then collected as baseband I/Q digital signals via two ADC channels and sent to the embedded controller. To further amplify the received signal, a low-noise amplifier (LNA) can be used. Since designing a low-noise amplifier (LNA) with low phase noise (NF) in the high-bandwidth terahertz frequency range is challenging, the platform utilizes an LNA at the IF stage, which operates at a frequency range of 1–6 GHz with a gain of 30 dB.

The baseband digital signal processing in the physical layer is implemented in a software-defined manner on the embedded controller PXIe-8881. At the transmitter, LabVIEW is used to construct user-defined information data into frames, which are processed through constellation mapping, pulse shaping, and other steps to generate baseband I/Q signals. These signals are then sent to the VST via the PXIe backplane. Similarly, at the receiver, LabVIEW is used to perform matched filtering, synchronization, and other processing on the baseband I/Q signals, which are then sent to a Python node as the training dataset for deep learning. The equalization training and equalization processes are conducted offline in Python. This baseband digital signal processing design greatly simplifies the design flow of different signal processing modules and enables a fast transition from simulation to experimentation in baseband digital signal processing.

### 3.2. Neural Network Architecture and Training Configuration

The proposed two-stage time-domain equalization framework is implemented using a standard LSTM module in PyTorch 2.1.1. No pre-trained weights or external models are used; all networks are trained from scratch using internal experimental data collected from the system described in Section 3.1.

Each equalizer stage uses a four-layer LSTM network with 128 hidden units in each layer, and dropout is applied between layers to improve generalization and prevent overfitting. The input dimension is 2, representing the real and imaginary parts of the received baseband signal; the output dimension is also 2, corresponding to the complex signal components after equalization.

The original dataset (210 GHz/2.1 m) contains 15,000 transmitted symbols, each representing two bits. The data are randomly divided into training, validation, and test sets in a ratio of 7:2:1. In addition, the test set also includes signals acquired at 310 GHz/1.5 m. After applying the data augmentation strategy described in Section 2.7 and the upsampling process described in Section 2.2 to the training set, the total number of training samples increases to approximately 168,000. Each sample consists of a transmitted sequence and a corresponding received waveform sequence that has been expanded according to the sampling factor. The test set and validation set apply the same sampling strategy when used, but no data augmentation is performed. It should be added that in order to compare the two-stage method and the two traditional equalizers more fairly, when we tested the versatility of the two-stage method (that is, using the 310 GHz test set), we retrained the two traditional equalizers because the two models had poor learning ability and versatility. Here, the two equalizers were trained with the same data preprocessing and data enhancement. The purpose is to ensure fairness (the two-stage time-domain equalization method is tested directly without retraining).

All input sequences are normalized and sliding-windowed following the preprocessing procedure described in Section 2.6. Training is supervised, using the transmitted symbols as target labels. For the first-stage equalizer, augmented data are used for training to improve its robustness under low-SNR conditions. For the second-stage equalizer, the model is trained directly on the output of the first-stage equalizer after downsampling, so no data augmentation is required.

**Figure 5:**
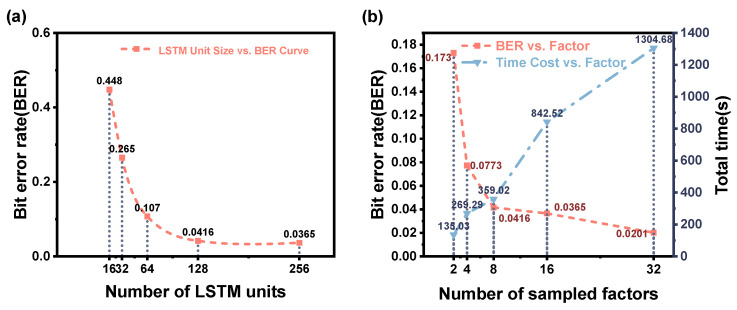
(**a**) BER performance of the two-stage equalization model with different numbers of LSTM units. As the number of units increases, the BER gradually decreases. However, beyond 128 units, the performance gain becomes marginal. Therefore, 128 is identified as the most cost-effective configuration based on our experimental results. (**b**) Comparison of BER and total computation time (including training and inference) under different up/downsampling factors. When the sampling factor is less than 8, the BER is significantly higher. When it exceeds 8, the BER improvement is negligible, but the total time increases substantially. Hence, a sampling factor of 8 provides the best trade-off between accuracy and efficiency. Both parameters were selected based on empirical evaluations to achieve a favorable balance between performance and computational cost during offline training.

Both stages are trained using the Adam optimizer with an initial learning rate of 0.001. A learning rate scheduling mechanism is used during training to allow the learning rate to be automatically adjusted based on the optimization progress. The batch size is set to 64, and a weight decay of $1\times 10^{-7}$ is used to apply mild L2 regularization. Although the maximum number of training epochs is set to 100, we use an early stopping strategy with patience set to 10 epochs, monitor the validation set loss to prevent overfitting and reduce unnecessary computation, and adjust the number of training epochs based on the training results. In addition, as shown in Figure 5, through experimental comparison, we selected the number of LSTM units and the sampling factor to achieve a good balance between performance and efficiency. All training is performed on an NVIDIA RTX 3060 GPU with 8 GB of memory. The entire training process is implemented and executed independently by the author without using any pre-training modules or external training assistance. The entire training process is handled offline. Table 1 summarizes the key experimental parameters and configurations used in this study.

## 4. Experimental Results and Analysis

In order to comprehensively evaluate the performance of the proposed two-stage equalization method, this section analyzes the signal characteristics before and after equalization in detail, and compares them with the traditional LSTM-based equalizer and Volterra nonlinear equalizer to evaluate the effectiveness of the three methods in suppressing nonlinear distortion. The BER of the three methods before and after equalization is shown in Table 2. It should be noted that due to factors such as nonlinearity, the dataset after data augmentation is more complex, so the Volterra equalizer and the traditional LSTM equalizer fail to learn the channel characteristics, so the BER is about 0.5. In the following discussion, we will compare the data before and after data augmentation to illustrate that our method has certain advantages before and after data augmentation, especially the latter; that is, when the nonlinear influence is large, it can still effectively equalize the received sequence. The final Losses of the three methods after data augmentation are Volterra: 0.112; traditional LSTM: 0.064; and two-stage LSTM equalization: 0.019. The Volterra equalizer uses a second-order kernel function commonly used in nonlinear FIR modeling, and its memory length is 15. At the same time, in order to better compare the two-stage time-domain equalization strategy with the traditional LSTM time-domain equalizer, we further evaluate its performance through the BER assisted by the signal waveform diagram and the constellation diagram, thereby qualitatively and quantitatively verifying the proposed method. Since the effect of the Volterra equalizer is similar to the conventional LSTM equalization result, the following discussion only compares the conventional LSTM with the two-stage time-domain equalization strategy.

**Figure 6:**
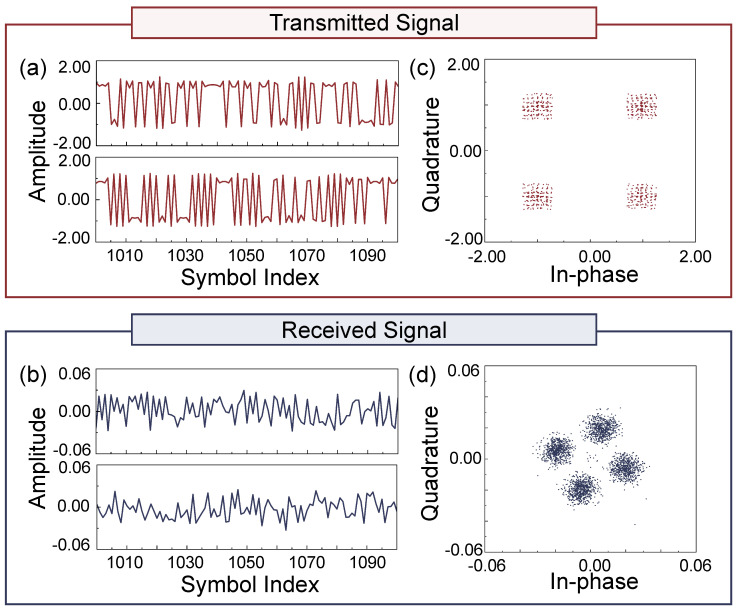
Experimental results over a 230 GHz, 2.1 m LoS THz channel. (**a**,**c**) Time-domain waveform and ideal constellation of the transmitted signal. (**b**,**d**) Received signal and its constellation after passing through the THz channel, showing significant distortion.

At the 230 GHz band with an LOS distance of 2.1 m, the received signals acquired after frame synchronization are shown in Figure 6b. Compared to the transmitted signals in Figure 6a, it is evident that the signals have been significantly affected by phase noise and hardware-induced nonlinearities during transmission. The demodulated BER reaches as high as 0.5072, further confirming that severe channel distortions have seriously degraded signal integrity. It is worth noting that, to clearly compare between the raw received signal, the signal processed by a conventional DL-based equalizer, and the signal processed by the proposed two-stage time-domain equalizer, only a portion of the signal waveform is displayed in the time-domain plots.

**Figure 7:**
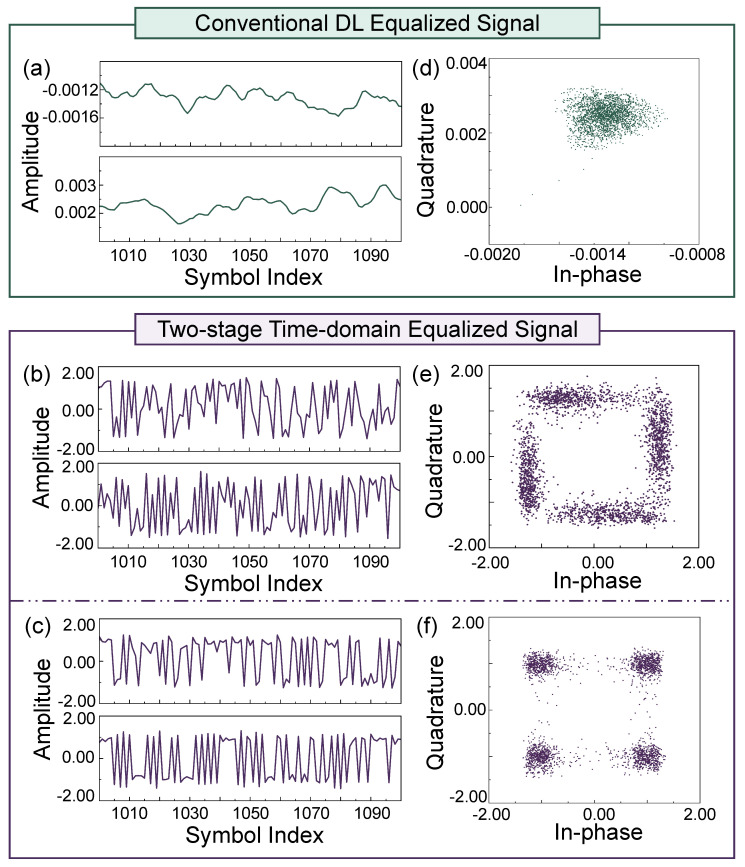
Experimental results over a 230 GHz, 2.1 m LoS THz channel. (**a**,**d**) Equalization result obtained by applying a conventional deep learning model directly to the raw signal, showing limited performance under the current setting. (**b**,**e**) Output after the first stage of the proposed two-stage time-domain equalization method. (**c**,**f**) Final equalized signal, demonstrating effective compensation for channel impairments and substantial restoration of the constellation integrity.

To highlight the advantages of the proposed method, the received signal is first directly processed by a traditional LSTM-based equalizer with the same architecture as the equalizer used in our model, without any upsampling. After equalization, the BER before and after data enhancement are 0.0022 and 0.4965, respectively. The results after data enhancement are shown in Figure 7a,d. From the time-domain waveform in Figure 7a, it can be seen that the signal amplitude fluctuates around zero. The constellation diagram in Figure 7d further shows that the model fails to learn the nonlinear distortion characteristics of the THz channel and the superimposed data enhancement processing to which the transmitted signal is subjected.

Subsequently, without modifying the LSTM architecture, we upsample the transmitted and received signals and apply the first-stage (global) equalization. The resulting time-domain waveforms are shown in Figure 7b. The waveforms after equalization show improved alignment with the original transmitted sequences in terms of both trend and amplitude. BER analysis indicates that the BER decreases to 0.1816 after the first-stage equalization, demonstrating the effectiveness of upsampling and the global equalizer in enhancing signal quality. However, the downsampled constellation diagram in Figure 7e reveals that while the overall constellation shape shows improved phase and amplitude recovery, some amplitude fluctuations remain. This suggests that although the first-stage equalizer is effective in correcting coarse signal distortions, it is insufficient in compensating for subtle nonlinear noise and residual distortions.

The downsampled signal is subsequently processed by the second-stage (local) equalizer. As shown in Figure 7c,f, the time-domain waveform after the second-stage equalization closely matches that of the transmitted signal, indicating a significant improvement in equalization performance. A comparison of the constellation diagrams before and after the second stage (Figure 7c vs. Figure 7f) reveals that the constellation points become more concentrated after the second-stage equalizer. The BER is further reduced from 0.1816 to 0.0398, demonstrating that the second-stage equalizer successfully compensates for most of the residual distortions and achieves the desired equalization effect. These results confirm that the proposed two-stage time-domain equalization method effectively implements a coarse-to-fine signal processing strategy. In addition, compared to conventional DL-based equalizers, the proposed method shows better adaptability across a wider range of channel conditions.

To further verify the robustness of the model, additional test data were collected at 310 GHz with a 1.5 m LOS channel. To prevent the two-stage model from memorizing training sequences rather than learning nonlinear channel characteristics, the new data were LDPC-encoded using an irregular parity-check matrix during transmission. The BER of the original, unprocessed signal is 0.012. After the first stage of equalization, the BER drops to 0.004, though slight distortions remain. Following the second stage of equalization, the BER is further reduced to 0.002, and the constellation points appear more concentrated, indicating improved signal quality. These results confirm that the two-stage equalizer can effectively enhance signal transmission under various frequencies and distances in complex THz channels.

Finally, we analyze three equalization methods that directly equalize the original collected signals without data enhancement processing. In the 230 GHz/2.1 m channel environment, the BER after Volterra equalization is 0.01104, the BER after traditional LSTM equalization is 0.0022, the BER after one-stage equalization of our method is 0.0013, and the BER after two-stage equalization is 0.0002. The problem reflected by such a BER is that only for the signals we actually collected, the two equalizers except for our method can show a relatively good effect, but the two-stage time-domain equalization method can still reduce the BER obtained by these two methods by one order of magnitude in the end. Combined with the signal equalization results after our data enhancement processing, we believe that our method can obtain slightly better results than the other two equalizers when the degree of channel nonlinearity is generally affected. When facing more complex nonlinearity and low SNR, our method can significantly lead the other two equalizers. Of course, this is the result obtained by sacrificing complexity. In the next section, we will discuss the impact of complexity on our method and the possibility of actual deployment.

In summary, the proposed two-stage time-domain equalization method mitigates signal distortion progressively, thereby improving transmission quality and reducing BER. This provides a feasible solution for signal equalization in THz communication systems, particularly in scenarios involving severe nonlinearity and signal attenuation. The proposed strategy demonstrates strong potential for future high-speed wireless communication systems.

### Complexity Analysis and Discussion

In this section, to evaluate the computational complexity of the proposed two-stage time-domain equalization framework, we use the analytical formula introduced in [43], which contains three indicators: the number of real-valued multiplications (RMs), the number of bit-level operations (BOPs), and the number of additions and bit shifts (NABSs) during inference. The calculation results are shown in Table 3.

The RM can be calculated as follows:

(11) $${\mathrm{RM}}_{\mathrm{LSTM}}=n_{s}n_{h}\left(4n_{i}+4n_{h}+3\right)$$

Here, $n_{s}$ denotes the sequence length (i.e., the number of time steps), $n_{h}$ denotes the number of hidden units per layer, and $n_{i}$ represents the input vector dimension. Compared to the notation used in Table 2, the notations $n_{s}$, $n_{h}$, and $n_{i}$ in this section correspond to *T*, *L*, and input feature dimensions, respectively. In the expression $n_{s}n_{h}\left(4n_{i}+4n_{h}+3\right)$, the three terms inside the parentheses represent the computational contributions from different parts of the LSTM cell: $4n_{i}$ corresponds to the input-to-hidden multiplications across four gates, $4n_{h}$ accounts for the hidden-to-hidden multiplications involving recurrent weights, and the constant 3 approximates the number of element-wise multiplications required by activation functions such as sigmoid, tanh, and Hadamard operations. Since these operations are performed across all time steps and hidden units, the total number of real-valued multiplications scales with $n_{s}n_{h}$.

The BOP can be calculated as follows:

(12) $$\begin{array}{cc} {\mathrm{BOP}}_{\mathrm{LSTM}}=\; & 4n_{s}n_{h}\cdot \mathrm{Mult}\left(n_{i},b_{w},b_{i}\right)\\ \; & +4n_{s}n_{h}\cdot \mathrm{Mult}\left(n_{h},b_{w},b_{a}\right)\\ \; & +3n_{s}n_{h}b_{a}^{2}\\ \; & +9n_{s}n_{h}\cdot \mathrm{Acc}\left(n_{h},b_{w},b_{a}\right) \end{array}$$

Here, $n_{s}$ represents the sequence length (the number of time steps), and $n_{h}$ represents the number of hidden units per layer, and $n_{i}$ represents the input vector dimension. The first term counts the bit-level multiplications between the input and weight matrices for each gate, with bit-widths $b_{w}$ and $b_{i}$. The second term performs the same for the hidden state and its weights, using $b_{a}$ as the activation bit-width. The third term estimates the cost from applying nonlinear functions. The last part accounts for the accumulations needed after all the multiplications.

The NABS can be calculated as follows:

(13) $$\begin{array}{cc} {\mathrm{NABS}}_{\mathrm{LSTM}}=\; & 4n_{s}n_{h}\left[n_{i}\left(X_{w}+1\right)-1\right]\cdot \mathrm{Acc}\left(n_{i},b_{w},b_{i}\right)\\ \; & +4n_{s}n_{h}\left[n_{h}\left(X_{w}+1\right)+1\right]\cdot \mathrm{Acc}\left(n_{h},b_{w},b_{a}\right)\\ \; & +6n_{s}n_{h}b_{a} \end{array}$$

Here, $n_{s}$ represents the sequence length (the number of time steps), $n_{h}$ represents the number of hidden units per layer, and $n_{i}$ represents the input vector dimension. The first part captures how many additions and shifts are needed for combining inputs and their weights, with $X_{w}$ representing whether weights are reused or sparse. The second part performs the same for the hidden state. The last term is a rough estimate of the extra additions and shifts inside the LSTM gates.

From the calculation results, the proposed two-stage time-domain equalization method has the highest computational complexity, which is about twice that of the conventional LSTM equalizer and much higher than the second-order Volterra equalizer. However, in terms of BER performance, the two-stage equalizer is significantly better than the conventional LSTM equalizer and the Volterra nonlinear equalizer.

This shows that the additional complexity introduced by this method is meaningful when dealing with nonlinear distortion that cannot be effectively suppressed by a single-stage equalizer. The inference time of the three equalizers measured in this study is as follows: Volterra = 0.844 s, traditional LSTM = 1.33 s, and two-stage equalization method = 2.37 s. Although the two-stage equalization method has a long inference time and currently has difficulty meeting the deployment requirements of real-time communication systems, since the method is not bound to the LSTM model, a shorter inference time can be achieved in the future by replacing the model and reducing the complexity of the algorithm.

Next, we would like to elaborate on the potential future potential of our model and why we believe it can be practically deployed. We believe that, given the current early stage of THz communication development and its limited transmission range [33], there is not yet an urgent demand for highly optimized channel equalizers. However, as discussed in the Introduction section, THz communications are affected by numerous impairments—such as water vapor absorption, atmospheric molecular attenuation, and hardware-induced nonlinearity—that necessitate the use of more advanced equalization techniques.

Our present work targets the equalization of nonlinear distortions introduced by THz channels. Experimental results indicate that, although our proposed method entails higher computational complexity, it consistently outperforms conventional nonlinear equalizers in both optimization accuracy and noise robustness. This validates the effectiveness of our two-stage approach in challenging channel environments.

Looking ahead, we believe that practical deployment of the proposed equalizer is feasible through future model and hardware optimizations. Importantly, our method is not inherently bound to the LSTM architecture; the current design can be readily replaced with more efficient models. As Freire et al. [43] have pointed out, by adopting lightweight alternatives and optimizing both the training and inference procedures, the path toward real-time implementation becomes realistic. This will be a key focus of our future work.

## 5. Conclusions

This study proposes a two-stage time-domain equalization method based on DL. This method adopts a global-to-local equalization strategy to effectively solve the factors that affect the communication quality in the terahertz channel, such as nonlinear distortion and random interference. In an indoor channel, the frequency is 230 GHz, the line-of-sight is 2.1 m, and experimental verification is carried out. Following two-stage time-domain equalization after data enhancement, the BER of the received signal is reduced from 0.5072 to 0.0398. In contrast, Volterra and traditional LSTM will be affected by the stronger nonlinear noise brought by data enhancement and cannot learn channel characteristics, which shows that the two-stage time-domain equalization has stronger anti-noise performance. In addition, the model shows a certain generalization ability. When applied to the newly collected received data generated by different transmission data under the same channel conditions (frequency is 310 GHz and line-of-sight is 1.5 m), the BER is reduced from 0.012 to 0.002, further demonstrating the versatility of the model. The above two-stage equalization process is an offline operation. The two-stage equalization method improves the stability and reliability of the communication quality of the terahertz communication system. However, due to the processing speed of the model, it is not currently applicable to real-time communication systems. Future research will focus on compressing and optimizing the model, as well as enhancing its adaptability to a wider range of channel environments, ultimately achieving real-time equalization across a variety of channel conditions.

## Figures and Tables

**Table 1:** Key experimental and training parameters.

Parameter	Value
System Configuration	
Modulation format	QPSK, 250 MHz bandwidth, 8 sps
Frame synchronization	32-symbol Zadoff–Chu, sliding correlation
Tx/Rx filters	Root-raised-cosine, roll-off = 0.25
Communication distance	1.5 m/2.1 m (Indoor LoS)
Air data rate	2 Gbps
VST output	2 GHz IF, −10dBm, 1 GS/s
LO frequency (MSG)	16.4 GHz, 9.5 dBm
Upsampling/Downsampling factor	8/8
Neural Network Configuration	
Equalizer input size	25 samples
Input/Output dimension	2/2 (I/Q components)
LSTM architecture	4 layers, 128 hidden units per layer
Dropout	Applied between LSTM layers
Loss function	Mean Square Error (MSE)
Optimizer	Adam
Initial learning rate	0.001 (with scheduling)
Weight decay	$1\times 10^{-7}$
Batch size	64
Max training epochs	100
Early stopping	Enabled (patience = 10, based on validation loss)
Data augmentation	Low-SNR noise (0–5 dB), applied to first stage only
Hardware Environment	
Training platform	NVIDIA RTX 3060 GPU
GPU memory	8 GB
Framework	PyTorch 2.1.1

**Table 2:** Comparison of equalization methods at 230 GHz and 310 GHz. All LSTM models use H=4 hidden layers, L=128 hidden units per layer, and T=25 input length. Two-stage deep learning equalizers are trained in two stages. Volterra equalizer uses a second-order kernel with a memory length of 15. Abbreviations: Config represents the network structure in the format of (*H*, *L*, *T*), where *H* is the number of hidden layers, *L* is the number of LSTM units, and *T* is the input sequence length. DA stands for data augmentation, and NDA stands for non-data augmentation.

Method	Config	BER (230 GHz (DA/NDA))	BER (310 GHz)
Traditional DL	(4, 128, 25)	0.4965/0.0022	0.0080
Two-stage DL (S1)	(4, 128, 25)	0.1816/0.0013	0.0040
Two-stage DL (Final)	(4, 128, 25)	0.0398/0.0002	0.0020
Volterra	(–, –, 25)	0.5003/0.0110	0.0107

**Table 3:** Comparison of inference complexity for different equalization methods. RM: Real-valued multiplication; BOP: bit-level operation; NABS: addition and bit shift.

Method	T	RM	BOP	NABS
Traditional DL	25	1.66 M	192.08 M	4.86B
Two-stage DL	25	3.32 M	382.16 M	9.72B
Volterra	25	0.0034 M	–	–

## Data Availability

Restrictions apply to the datasets. The datasets provided in this article are not easily accessible because the experimental setup involves a custom LabVIEW control program and a deep learning-based signal processing model, and the data are part of other ongoing research. To access the datasets, please contact the authors at 2230602063@cnu.edu.cn.
